# Burden and Risk Factors of Postpartum Depression in Southwest Saudi Arabia

**DOI:** 10.3390/jcm12103444

**Published:** 2023-05-13

**Authors:** Safar Abadi Saeed Al-Saleem Alshahrani, Norah Fayz Al-Saleh, Aesha Farheen Siddiqui, Shamsun Nahar Khalil, Mohammed Abadi Alsaleem, Hayfa A. AlHefdhi, Sahar Awad Al-Qadi, Abdullah Ahmad Asiri

**Affiliations:** 1 Family and Community Medicine Department, College of Medicine, King Khalid University, Abha 62529, Saudi Arabia; 2 Ministry of Health, Abha 61521, Saudi Arabia

**Keywords:** post-partum, depression, post-natal, Edinburgh postnatal depression scale, Saudi Arabia

## Abstract

Objectives: The burden of postpartum depression (PPD) is significant because it remains unrecognized, and it not only affects the mother adversely but also has a negative consequence on the family life and the development of the infant. The aim of the study was to measure the prevalence of PPD and identify the risk factors of PPD among mothers attending the well-baby clinic of six Primary Health Care centers in Abha city, Southwest Saudi Arabia. Materials and Methods: A total of 228 Saudi women having a child aged between two weeks to one year were recruited in the study by using a consecutive sampling technique. The Arabic version of the Edinburgh Postnatal Depression Scale (EPDS) was used as a screening tool to measure the prevalence of PPD. The mothers were also inquired about their socio-demographic characteristics and risk factors. Results: The prevalence rate of postpartum depression was 43.4%. Family conflict, and lack of support by spouse and family during pregnancy were found to be the strongest predictors of developing PPD. Women who had reported family conflict were at six times higher risk for developing PPD compared to those who did not have a family conflict (aOR = 6.5, 95% CI = 2.3–18.4). Women who reported a lack of spousal support during pregnancy encountered 2.3 fold increased risk for PPD (aOR = 2.3, 95% CI = 1.0–4.8) and women who had not received family support during pregnancy period were more than three times (aOR = 3.5, 95 % CI 1.6–7.7) likely to experience PPD. Conclusion: The risk of PPD among Saudi postnatal women was high. PPD screening should be an integral part of postnatal care. Awareness of women, spouses and families about potential risk factors can be a preventive strategy. The early identification of high-risk women during the antenatal and post-natal period could help to prevent this condition.

## 1. Introduction

Pregnancy is an enormous change in a woman’s life. Having a baby can be a very exciting time, at the same time, the emotional, hormonal, and physical changes a woman bears during this process can be hard and stressful, causing her to feel sad, anxious, afraid, and confused. Most women who experience these emotional changes are able to recover quickly. For some women, however, these feelings do not go away and may get worse.

In the Diagnostic and Statistical Manual of Mental Disorders (DSM–5), postpartum depression is considered when a patient has a major depressive episode along with the peripartum onset. By definition, it is defined as a major depressive episode with the onset of pregnancy or within four weeks of delivery [1]. Three types of illness have been classified based on the symptoms; baby blues, postpartum depression, and postpartum psychosis [1]. Baby blues that begins within the firstweek post-delivery is the least severe form of depression affecting between 13.7% to 76.0% of postpartum women [2]. Baby blues are described by symptoms, such as mood changes, dysphoria, anxiety, sleep disturbance, lack of appetite, irritability, tearfulness, agitation, lack of confidence, and feelings of being overwhelmed. Persisting for more than two weeks indicates that the woman may have advanced into postpartum depression [1].

The global prevalence of PPD varies between 10 and 33% [2,3,4,5,6], while in the middle-east region, it ranges from 17.6% to 51% [7,8,9,10,11,12]. Within Saudi Arabia, PPD affects 14–38.5% women in their post-partum period [13,14,15,16]. PPD usually occurs from four to twelve weeks after birth but can happen up to a year after delivery. Untreated PPD can lead to chronic depression and other complications, such as anxiety, poor quality of life, poor social and sexual relationships, risky behaviors, and suicidal ideation [17]. In addition to directly influencing the emotional well-being of mothers, postpartum depression has also been shown to affect mother–infant bonding and infant behavior. Children’s cognitive and emotional development can be significantly affected by postpartum depression [18,19,20].

A large body of evidence has consolidated the factors that can place mothers at risk for post-partum depression. These include prior history of depression, family history of depression, unemployment, low level of education, preterm birth, cesarean delivery, unplanned pregnancy, hormonal changes during pregnancy, lack of spousal support and insufficient family support, marital conflicts, stressful life events, such as involvement in violent relationships, and traumatic experiences [6,12,21,22].

Most pregnant women do not know about PPD and do not seek help, even when having experienced similar problems in previous pregnancies [18,23]. Postpartum women need early screening and quick access to mental healthcare [24,25]. Very limited studies have been carried out on this issue in Saudi Arabia. Particularly in the Southern region, no such study has been conducted. Hence a study on postpartum depression and associated risk factors was urgently needed to inform the current situation to health care providers and policymakers. Therefore, the aim of this study was to estimate the prevalence of postpartum depression and to identify the risk factors associated with PPD which would improve our understanding of the picture of PPD and also inform prevention and intervention strategies.

## 2. Methods

### 2.1. Study Place and Population

This cross sectional study was conducted in Abha city, the capital of Aseer Region in Saudi Arabia. The study period was from August to December 2019. The study participants comprised of post-partum Saudi women between 18–48 years of age with a child between 2 weeks to one year of age. Non-Saudi mothers and mothers having a child less than two weeks of age and more than one year of age were excluded from the study. The women were recruited from well-baby clinics at six primary health care centers randomly chosen from twelve primary health care centers in Abha city.

### 2.2. Sample Size and Sampling Technique

The sample size was calculated by using the statistical formula n = Z^2^pq/d^2^ [26]. Here, Z is the confidence limit (1.96) at a 95% level. “p” is the prevalence rate of postpartum depression (33.2%) in a Saudi Arabia study [21], “q” is (1 − p) that is the proportion of women who did not have postpartum depression, and “d” is the acceptable standard error (6%). The calculated sample size was 236. Consecutive sampling technique, that is participants were recruited on a first come basis, continuously till the desired sample size was reached. A total of 236 participants were approached. Due to incomplete information, eight questionnaires were excluded and as a result, the final sample size remained at 228.

### 2.3. Data Collection Tool

Arabic version of the Edinburgh Postnatal Depression Scale [27] was used as a screening tool to identify post-partum depression among the study subjects. The EPDS is a 10-item self-report questionnaire designed specifically for the detection of depression in the postpartum period [28]. It has been validated and translated into more than 20 languages, including Arabic. It is proven to be an effective screening tool and has been shown to have a sensitivity of 95% and specificity of 93%. Responses are scored 0–3 indicating the severity of manifestations with a minimum score of 0 and a maximum score of 30. The EPDS has a cutoff of 10 to signify probable depression [27]. Mothers with EPDS scores of more than 10 were classed as having depression. An increased score indicates the increasing severity of depression. A questionnaire covering the socio-demographic information and risk factors for PPD was developed and included with EPDS.

### 2.4. Study Procedure

The researcher provided a brief explanation about the objective of the study to the participants before the interview. The participants were assured of the confidentiality of all information. Voluntary verbal consent was obtained from participants before the interview. The study proposal was approved by the King Khalid University research ethical committee. (Reference #: REC: 2016/05/14). At the end of the study, mothers identified with high EPDS scores were advised to consult a psychiatrist for confirming the diagnosis and treatment.

### 2.5. Data Analysis

Data entry and analysis were conducted by using statistical software package SPSS version 22.0 [29]. Categorical data were presented as frequency and percentages and continuous data were presented as mean ± standard deviation. Univariate analysis was initially performed using Chi-square test or Fisher exact test to find the association of various risk factors with postpartum depression. Multiple logistic regression analysis was conducted using only the independent variables that were significant in the univariate analysis. A *p*-value less than 0.05 was considered statistically significant. Adjusted odds ratios in multiple logistic regression were measured with a 95% Confidence Interval.

### 2.6. Findings

The study participants comprised post-partum women between 18–48 years of age with a mean age of 30.9 ± 7.0 years. One hundred and ten women (48.2%) were less than 30 years of age, while 118 (51.8%) women were more than thirty years of age. The monthly income of 42.5% of women was between SR. 5000–10,000 and 37.7% was more than SR. 10,000. Almost all women (98.2%) were non-smokers. The pregnancy-related characteristics of the study group revealed that three in four were multi-gravid. More than half of the women (54.8%) reported that their last pregnancy was planned. No complication in last pregnancy was reported by 82.5% of women. Gestational diabetes was reported by 22 (9.6%). Most of women (about 90%) did not report any complications during or after delivery for themselves or the neonate. Sex of last child revealed an almost equal sex ratio. Few mothers delivered twins (3.5%). Current psychiatric illness was reported by 12 respondents (5.3%), while 11.8% reported a positive family history of psychiatric disease. Existing family conflict was reported by 1 in 6 respondents, while the past family conflict was reported by almost a third of the women. Three out of every four women reported support from their husband during pregnancy, and a little higher (76.8%) reported support by family in pregnancy.

**Figure 1:**
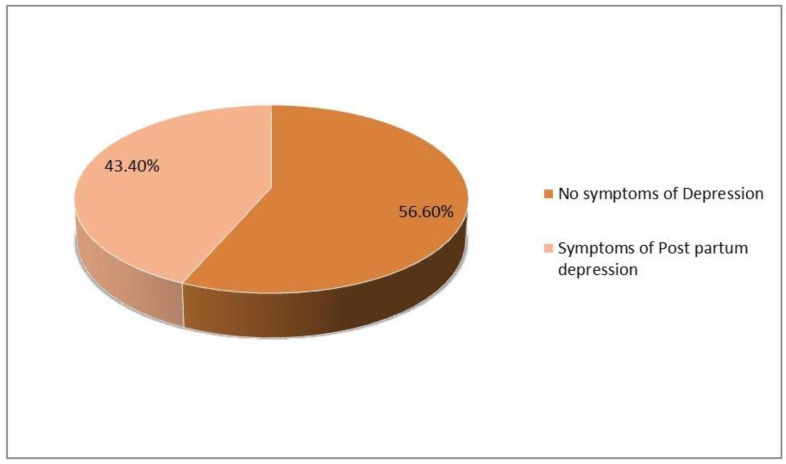
Proportion of respondents with post-partum depression.

**Figure 2:**
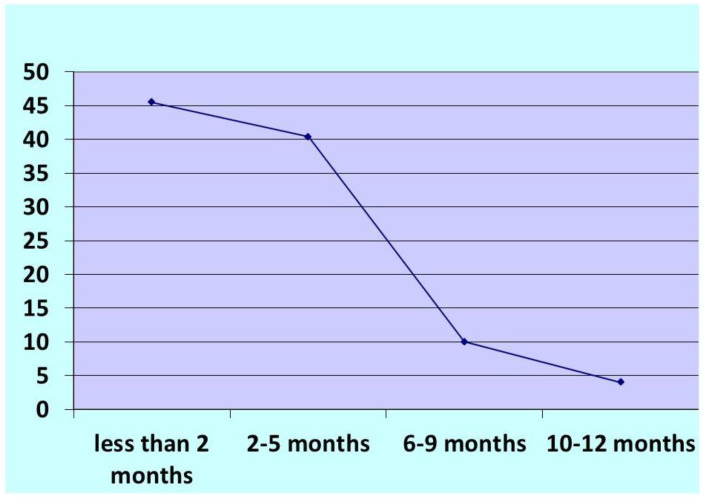
Percentage distribution of postpartum depression cases by time since delivery.

Figure 1 presents the proportion of post-partum depression among the respondents. Based on the Edinburgh screening (EPDS) instrument, 99 (43.4%) mothers had depressive symptoms. Regarding the time since delivery for women with depressive symptoms, almost 46% of women were within 2 months of delivery, 40% were within 2–5 months of delivery, 9.5% between 6–9 months, and only about 4 % of cases between 10–12 months of delivery (Figure 2).

Table 1 presents the association of postpartum depression with socio-demographic factors. A larger proportion of women with depression belongs to older ages (57.6%) and had a higher education (87.9%). Postpartum depression was more common in non-working women (59.6%), lower family income group (68.7%) and married women. However, none of these factors showed any significant association with postpartum depression.

Table 2 demonstrates the association of obstetric factors with postpartum depression. Multi-gravid women had significantly more postpartum depression than primigravid women (cOR = 2.0, 95%CI 1.1,3.8; *p* = 0.03). All other factors, such as planned pregnancy, outcome of pregnancy, sex of child, time, and mode of delivery, complication during or after delivery, twins or gestational diabetes did not show any significant association with postpartum depression.

The association of psychosocial characteristics with postpartum depression is presented in Table 3. Women with existing psychiatric illness (cOR = 4.2, 95% CI 1.1,5.9; *p* = 0.024) and those with family history of psychiatric illness (cOR = 3.5, 95% CI 1.5,8.6; *p* = 0.003) had significantly more postpartum depression than those without. Significant association was also observed for those women who reported existing (cOR = 8.3, 95% CI 3.5, 19.8; *p <* 0.001) or past family conflict (cOR = 2.6, 95%CI 1.4,4.6; *p* = 0.001), and lack of support from spouse (cOR = 4.4, 95% CI 2.3,8.5; *p <* 0.001) and family during pregnancy (cOR = 5.3, 95% CI 2.6,10.6; *p <* 0.001).

Multivariate regression analysis was performed using only those variables that were found significant in univariate analysis shown in Table 4. Existing family conflict, lack of support by spouse, and lack of family support during pregnancy were identified as predictors for PPD. Women who had reported existing family conflict were at six times higher risk for developing postpartum depression compared to those women who did not (aOR = 6.5, 95% CI = 2.3,18.4). Women who did not receive care or support from their spouse during pregnancy faced more than two-fold increased risk for postpartum depression (aOR = 2.3, 95% CI 1.0,4.8) compared to those who were supported by their spouses. Women who had not received family support during the pregnancy period were more than three times more (aOR = 3.5, 95 % CI 1.6–7.7) likely to experience postpartum depression.

## 3. Discussion

Postpartum depression (PPD) is a major depressive disorder and is recognized as an important public health problem for women of reproductive age. It has long-term implications for the mother and infant. Untreated PPD causes maternal distress that affects parenting, maternal bonding, and also the infant’s emotional, cognitive, and behavioral development [20,28,29]. It has been repeatedly reported that early diagnosis would help in implementing preventive strategies to prevent the worsening of the problem. As is evident from studies, mothers fail to recognize the symptoms and it is imperative that healthcare providers detect early any symptoms of depression in recently delivered mothers [18]. Hence, this study on postpartum depression among Saudi women would help in informing the current situation to the community and health care providers.

The worldwide prevalence of PPD ranges from 10–33% [3,4,5,6] while the prevalence of PPD in the middle-east region ranges from 17.6% to 51% [7,8,9,10,11,12]. Prevalence rates differ due to cross-cultural and social factors [21,30]. In a study from India, factors in India, such as poor living conditions, family disputes, crises, financial issues, more children to take care of, and fewer work opportunities, are the factors influencing postpartum depression [31]. Urban, low-income, and married mothers have different risk factors and higher rates of PPD than their more affluent counterparts in Midwest, North America [32].

Within Saudi Arabia, wide differences are reported in the prevalence of PPD ranging from 14–38.5% [13,14,15,16]. The current study observed a higher prevalence (43.4%). This difference can be explained by the difference in study method used, diagnostic criteria, the cutoff point for screening, and time of screening and location of the study. The cutoff point for screening was 2–6 months in the study by Al-Asoom [13] and 2–3 months by Al-Harbi [15], whereas it was 2 weeks–1 year in our study. Similarly, in the study by Al-Mudayfar [14], the sample size was 1200, and cutoff point was also different from our study.

A considerable body of research has examined multiple risk factors to be associated with postpartum depression. These include socio-demographic, obstetric, and psychosocial factors. In the current study, no associations were observed with any of the socio-demographic factors. This is in resonance with other studies in Saudi Arabia [13,14,15,16].

Maternal health during pregnancy and some obstetric factors increase the risk of development of post-partum depression [33]. Studies in Saudi Arabia reported that mothers who have poor health during pregnancy premature delivery, unplanned pregnancy, and delivery by cesarean section have a significant risk for subsequently developing postnatal depression [13,14,15,16]. Surprisingly, no such association was observed in the current study except multigravidity. Association with gravidity could be due to stress related to successive pregnancies and caring for many children at the same time.

The psychosocial factors that increase mothers’ risk for postpartum depression include prior history of depression, family history of depression, lack of family support, marital conflict, stressful life events, such as involvement in violent relationships, and traumatic experiences [6,12,21,22]. Research from the region consistently reported stressful life events and traumatic experiences, such as current family conflict, a non-supportive spouse, and a non-supportive family during pregnancy, as predictors of PPD [15,34,35]. This is also reflected in the current study. We identified family conflict, non-supportive spouse, and non-supportive family as predictors of PPD in our study group. Many women experience stress during pregnancy and motherhood. This stress is compounded by a lack of support from a spouse and family. Support provided by the partner as well as by family acts as a buffer against the difficulties in the transition to motherhood and helps to safeguard women’s mental health [33].

Screening for depression in the early postpartum period helps in early detection and prompt treatment of PPD. In Ref. [24], it is recommended that patients who screen positive and meet diagnostic criteria for PPD should receive prompt treatment [25]. A majority of women do not recognize the symptoms of depression and fail to seek help for PPD [18,23]. Therefore, primary care physicians should screen for depression at every opportunity early in the postpartum period. It is expected that the findings of the study can motivate health care providers who work with pregnant women to include assessments of these factors in their routine examinations and to improve the early identification and management of this condition.

## 4. Strengths and Limitations of the Study

Our study is the first of its kind to examine postpartum depression in this region of Saudi Arabia. It provides a picture of the prevailing situation in this area and shows the size of the problem and the main priorities to be focused on maternal health programs for this population of Saudi Arabia. Despite this, the study has also a number of limitations. Firstly, this study sample selected six urban PHCCs and consecutive sampling was adopted. Thus, the results of this study cannot be generalized to all women from Saudi Arabia. Secondly, the recall bias could not be avoided due to self-reported questionnaire, and all data were extracted from maternal recall. Finally, although this study explored a vast number of factors associated with PPD, there are many other factors, such as hormonal effect, post-natal anemia, marital discord, intimate partner violence, and lack of social support, cultural factors, and other physical health problems that may affect the associations and need exploration.

## 5. Conclusions

Our study provides evidence that a substantial proportion of Saudi women experience post-partum depression. Family conflict, non-supportive spouses, and non-supportive families act as precursors of PPD. Increasing the awareness of women, men and family members about potential risk factors of PPD can be a preventive strategy. Healthcare providers need to be aware of and receive appropriate training on dealing with the psychosocial aspects of women’s health. PPD screening should be an integral part of postnatal care as early identification of high-risk women could help to prevent post-partum depression and its complications.

## Figures and Tables

**Table 1:** Association of socio-demographic variables with postpartum depression.

Variables		Post-Partum Depression			Unadjusted OR [95%CI]
	Group	No	Yes	*p* Value	
Age	≤30 years	68 (52.7%)	42 (42.4%)	0.123	1.5 [0.89,2.6]
	≥30 years	61 (47.3%)	57 (57.6%)		
Education	Intermediate and below	11 (8.5%)	12 (12.1%)	0.372	0.67 [0.28,1.6]
	Secondary and above	118 (91.5%)	87 (87.9%)		
Occupation	Non-working	78 (60.5%)	59 (59.6%)	0.894	1.0 [0.61,1.8]
	Working	51 (39.5%)	40 (40.4%)		
Income	≤10,000 SR	74 (57.4%)	68 (68.7%)	0.80	0.61 [0.35,1.1]
	>10,000 SR	55 (42.6%)	31 (31.3%)		
* Marital status	Married	127 (98.4%)	95 (96.0%)	0.24	2.6 [0.48,14.9]
	Divorced	02 (1.6%)	04 (4.0%)		
* Smoker	Yes	02 (1.6%)	02 (2.0%)	0.789	0.76 [0.11,5.5]
	No	127 (98.4%)	97 (98.0%)		

* Fischer’s exact test.

**Table 2:** Association of obstetric factors with postpartum depression.

Variable		Post-Partum Depression			Unadjusted OR [95%CI]
	Group	No	Yes	*p* Value	
Gravida	Primi	38 (29.5%)	17 (17.2%)	0.03	2.0 [1.1,3.8]
	Multi	91 (70.5%)	82 (82.8%)		
Whether Pregnancy was planned	Yes	75 (58.1%)	50 (50.5%)	0.25	1.3 [0.81,2.3]
	No	54 (41.9%)	49 (49.5%)		
Complication in pregnancy	Yes	27 (20.9%)	13 (13.1%)	0.12	1.7 [0.85,3.6]
	No	102 (79.1%)	86 (86.9%)		
Gestational diabetes	Yes	14 (10.9%)	8 (8.1%)	0.48	1.3 [0.55,3.4]
	No	115 (89.1%)	91 (91.9%)		
Mode of delivery	Normal	86 (66.7%)	62 (62.6%)	0.52	1.2 [0.69,2.1]
	C/S	43 (33.3%)	37 (37.4%)		
Complication during delivery	Yes	08 (6.2%)	09 (9.1%)	0.41	0.66 [0.25,1.8]
	No	121 (93.8%)	90 (90.9%)		
Postpartum complication	Yes	10 (7.8%)	10 (10.1%)	0.53	0.75 [0.29,1.8]
	No	119 (92.2%)	89 (89.9%)		
Neonatal complication	Yes	09 (7.0%)	11 (11.1%)	0.27	0.61 [0.24,1.5]
	No	120 (93.0%)	88 (88.9%)		
Twin delivery	Yes	06 (4.7%)	02 (2.0%)	0.28	2.3 [0.47,11.9]
	No	123 (95.3%)	97 (98.0%)		
Pregnancy outcome	Live	126 (97.7%)	93 (93.9%)	0.07	2.7 [0.66,11.1]
	Not live	03 (2.3%)	06 (6.1%)		
Sex of child	Male	61 (47.3%)	54 (54.5%)	0.27	0.75 [0.44,1.26]
	Female	68 (52.7%)	45 (45.5%)		

**Table 3:** Association of psychosocial characteristics with postpartum depression.

Variable		Post-Partum Depression			
	Group	No	Yes	*p* Value	Unadjusted OR [95%CI]
Psychiatric disease	No	126 (97.7%)	90 (90.9%)	0.024	4.2
	Yes	03 (2.3%)	09 (9.1%)		[1.1,5.9]
Family History of Psychiatric disease	No	121 (93.8%)	80 (80.8%)	0.003	3.5
	Yes	08 (6.2%)	19 (19.2%)		[1.5,8.6]
Existing family conflict	No	122 (94.6%)	67 (67.7%)	˂0.001	8.3
	Yes	07 (5.4%)	32 (32.3%)		[3.5,19.8]
Past family conflict	No	100 (77.5%)	56 (56.6%)	0.001	2.6
	Yes	29 (22.5%)	43 (43.4%)		[1.4,4.6]
Support by Spouse	No	17 (13.2%)	40 (40.4%)	˂0.001	4.4
	Yes	112 (86.8%)	59 (59.6%)		[2.3,8.5]
Support by family	No	14 (10.9%)	39 (39.4%)	˂0.001	5.3
	Yes	115 (89.1%)	60 (60.6%)		[2.6,10.6]
Living with spouse only	No	50 (38.8%)	45 (45.5%)	0.189	0.75
	Yes	79 (61.2%)	54 (54.5%)		[0.45.1.2]

**Table 4:** Multivariate logistic regression analysis for the predictors of PPD.

Variable	Group	Edinburgh Score		aOR *	95%CI	*p* Value
		No PPD	Yes PPD			
Psychiatric Disease	No	126 (97.7%)	90 (90.9%)	3.53	Reference	
	Yes	03 (2.3%)	09 (9.1%)		[0.74,16.7]	0.543
Family History of Psychiatric disease	No	121 (93.8%)	80 (80.8%)	1.80	Reference	
	Yes	08 (6.2%)	19 (19.2%)		[0.63,5.1]	0.675
Existing family conflict	No	122 (94.6%)	67 (67.7%)	6.5	Reference	˂0.001
	Yes	07 (5.4%)	32 (32.3%)		[2.34,18.4]	
Past family conflict	No	100 (77.5%)	56 (56.6%)	0.82	Reference	0.891
	Yes	29 (22.5%)	43 (43.4%)		[0.37,1.8]	
Support by Spouse	No	17 (13.2%)	40 (40.4%)	2.3	[1.2,4.8]	˂0.001
	Yes	112 (86.8%)	59 (59.6%)		Reference	
Support by family	No	14 (10.9%)	39 (39.4%)	3.5	[1.6,7.7]	
	Yes	115 (89.1%)	60 (60.6%)		Reference	˂0.001
Gravida	Primi	38 (29.5%)	17 (17.2%)	0.26	Reference	
	Multi	91 (70.5%)	82 (82.8%)		[0.21,1.8]	0.345

* aOR-Adjusted Odd Ratio.

## Data Availability

The data is available with the first author.
